# Energy and Environment Performance of Resource-Based Cities in China: A Non-Parametric Approach for Estimating Hyperbolic Distance Function

**DOI:** 10.3390/ijerph17134795

**Published:** 2020-07-03

**Authors:** Yao Hu, Tai-Hua Yan, Feng-Wen Chen

**Affiliations:** School of Economics and Business Administration, Chongqing University, Chongqing 400030, China; 20150201014@cqu.edu.cn

**Keywords:** hyperbolic distance function, HDF, green development, energy and environmental productivity, resource-based city, data envelopment analysis (DEA)

## Abstract

Scientific determination of energy and environmental efficiency and productivity is the key foundation of green development policy-making. The hyperbolic distance function (HDF) model can deal with both desirable output and undesirable output asymmetrically, and measure efficiency from the perspective of “increasing production and reducing pollution”. In this paper, a nonparametric linear estimation method of an HDF model including uncontrollable index and undesirable output is proposed. Under the framework of global reference, the changes of energy environmental efficiency and productivity and their factorization of 107 resource-based cities in China from 2003 to 2018 are calculated and analyzed. With the classification of resource-based cities by resource dependence (RD) and region, we discuss the feature in green development quality of those cities. The results show that: (1) On the whole, the average annual growth rate of energy and environmental productivity of resource-based cities in China is 2.6%, which is mainly due to technological changes. The backward of relative technological efficiency hinders the further growth of productivity, while the scale diseconomy is the main reason for the backward of relative technological efficiency. (2) For the classification of RD, the energy and environmental efficiency of the high-dependent group are significantly lower than the other two, and the growth of productivity of the medium-dependent group is the highest. (3) In terms of classification by region, the energy and environmental efficiency of the eastern region is the highest, and that of the middle and western regions is not as good as that of the eastern and northeastern regions. The middle region shows the situation of “middle collapse” in both static efficiency and dynamic productivity change, and the main reason for its low productivity growth is the retreat of relatively pure technical efficiency. This conclusion provides practical reference for the classification and implementation of regional energy and environmental policies.

## 1. Introduction

With the shortage of energy and the increasingly severe environmental pollution in China, the issue of green development has aroused a growing attention from scholars. Implementing the green transformation for China entails very serious operations, especially for those resource-based cities [1]. As an important cornerstone of China’s modern industrial system, resource-based cities have attracted a large number of heavy chemical enterprises with high energy consumption and high pollution because of their raw material advantages [2]. They are not only the main suppliers of industrial raw materials and energy, but also the main undertakers of resource consumption and environmental damage. At present, China’s economy is in a critical period of transformation: upgrading industrial structure and moving towards a high-quality development level. Sustainable growth is one of the important internal requirements to achieve high-quality economic development. Therefore, the green transformation of resource-based cities is not only an important starting point for the transformation to high-quality development from a national level, but also an inevitable requirement for the sustainable development of cities themselves [3]. The key lies in improving energy and environmental productivity, consuming less energy and discharging lower level pollutants as much as possible to achieve higher economic growth. Thus, in this context, scientifically measuring the energy environmental efficiency and productivity of resource-based cities, and exploring the linkages between energy consumption, economic output, and negative environmental impacts is of great significance to practice the transformation of green development mode, promote the sustainable development of resource-based cities, and encourage high-quality economic development.

Since the concept of total factor energy efficiency was put forward by Hu and Wang [4], the measurement of energy efficiency in relevant researches has gradually changed from single factor indicators, such as energy intensity, to total factor indicators, in which comprehensive input factors such as capital and labor are also concerned. The main idea is to use the samples to build the technology frontier under the total factor framework, and therefore the gap between the actual input and potential minimum input of energy reflects inefficiency. There are often two methods to measure the inefficiency or efficiency: the stochastic function analysis (SFA) and data envelopment analysis (DEA); the latter is increasingly used in recent researches because of its deterministic frontier constructed and non-parametric characteristics, as well as its easy combination with the Malmquist index, by which the productivity can be evaluated further. At the same time, Chung et al. [5] provided a viable approach to regard other bad outputs, such as environmental indicators, as undesirable outputs in the total factor framework, illustrating with the directional distance function (DDF) that it is feasible to measure energy environmental efficiency.

Combining different kinds of DEA and Malmquist models, a large number of papers have explored energy environmental efficiency and productivity, also called green efficiency and productivity [6] or ecological efficiency and productivity [7]. For environment quality assessment, Iram et al. [8] utilized DEA to determine the efficiency of energy usage and its role in carbon dioxide emissions and economic-environmental efficiency for the economies of some Organisation for Economic Co-operation and Development (OECD) countries from 2013 to 2017. Bi et al. [9] used slack-based DEA to investigate the relationship between fossil fuel consumption and the environmental regulation of China’s thermal power generation. Song et al. [10] proposed a Ray slack-based model to evaluate provincial environmental efficiencies in China from 2004 to 2012. Halkos et al. [11] integrated radial and non-radial efficiency measurements in DEA using the hybrid measure by considering good and undesirable outputs as separable and non-separable to estimate the efficiency of the power generation sector in the USA with the DEA window analysis. Wang and Feng [12] evaluated the performance of China’s energy, environmental, and economic efficiency and its productivity growth therein from 2002 to 2011 by a developed slack-based measure based on global data envelopment analysis. Yang and Wei [13] applied game cross-efficiency DEA to analyze the urban total factor energy efficiency of 26 Chinese prefectural-level cities from 2005 to 2015 under environmental constraints. Regarding research on resource-based cites, Li et al. [14] used DEA model to evaluate the transformation development efficiency of Jiaozuo from 1999 to 2013. Based on the 2012 data from 116 resource-based cities in China, Li and Dewan [15] measured efficiency in resource-based cities by using the Super-slack based model (SBM) model and analyzed its influence. Yan et al. [16] analyzed the spatial variation in energy efficiency of 104 resource-based cities in China based on Super-SBM from an energy-economy-environment perspective. Tian et al. [17] utilized the modified meta-frontier Epsilon-based measure (EBM) model to measure the CO_2_ emission efficiency, comparing the performance between the Yangtze River Economic Belt (YREB) and non-YREB.

However, the models used in the aforementioned papers can hardly deal with the desirable output and the undesirable output asymmetrically in the efficiency measurement function. The hyperbolic distance function (HDF) is the exact one to solve this problem, but its non-parametric optimization model is non-linear, which is very difficult to solve especially with variable return scale assumption. Although Fare et al. [18], the authors of this model, provide a rough estimation method, there are large errors in the results. In relevant studies [19,20,21,22]), the measurement of HDF is mainly solved by the parameter method, SFA, which is constructed based on transcendental logarithm function for regression estimation, depending on the model setting and hypothesis. That can also hardly analyze the scale effect. Fare et al. [23] further proposed a linear iterative algorithm to estimate HDF using DDF, which improved the estimation accuracy under the original HDF model on the premise of satisfying the operational properties under the non-parametric DEA framework, but did not discuss the situation of including uncontrollable indicators and undesirable output. Zhang and Jiang [24] argued that the relevant research on energy efficiency pointed out that some scholars failed to eliminate the influence of other factors such as capital and labor on the measurement of total factor energy efficiency, by which the measured efficiency can be also called both the total factor energy efficiency and the total factor capital and labor efficiency. That is to say, it failed to control the inputs other than energy in the model, which may lead to confusion on the concept.

Few studies have focused on the energy environmental efficiency of resource-based cities. Yu et al. [25] applied a three-step material flow analysis (MFA) method to analyze the resource utilization efficiency, but only used Chengde in Hebei Province as a case. Li and Dewan [15] used cross-sectional data for 2012 to measure the efficiency of China’s 116 resource-based cities but lacked analysis of time series. Wei et al. [26] employed DEA model and Malmquist productivity index to measure the urban efficiencies of only 24 typical resource-based cities in China from 2000 to 2008. Related similar studies include those by Yan et al. [16] and Liu and Meng [27].

Most resource-based regions have suffered a lot from a series of development problems caused by the “resource curse”, such as experiencing long-term weak growth of gross domestic product (GDP), economic vulnerability, growing income gap in society, and more and more serious political corruption [28,29,30,31,32]. In resource curse theory, resource dependence (RD) is the key conception to exert negative influence upon economic development, which reflects the importance of resource exploitation or resource industry in economic growth. In order to distinguish resource abundance, RD is commonly interpreted as the output elasticity coefficient of economic growth to resource input in the production function including resource factors [33]. According to the “resource curse”, the energy environmental performance of cities may be affected by RD, implying cities with different RD should be treated differently when making policy. So how dose RD affect energy environmental efficiency in China?

Thus, in this paper, in order to measure the energy environmental efficiency and productivity changes scientifically and accurately, we propose a nonparametric linear estimation method of HDF model including uncontrollable index and undesirable output. To further provide empirical support for better policy-making for governments, by focusing on 107 resource-based cities in China, we divide the cities into three and four categories from the perspective of RD and region and discuss the performance of different types of cities.

The paper next is organized as follows: the research methodology applied in this research is illustrated in Section 2, material is presented in Section 3, followed by an empirical study in Section 4, and the conclusions are presented in Section 5.

## 2. Research Methodology

### 2.1. HDF Model with Environmental Technology

Assuming the production technology *T* based on environmental technology which transforms a series of input vectors $x\in {\mathbb{R}}_{+}^{M}$ into the desired output vectors $g\in {\mathbb{R}}_{+}^{S}$ and the undesired output vectors $b\in {\mathbb{R}}_{+}^{L}$, the production possibility set P(x) can be defined by

(1) P(x)={(g,b)|(x,g,b)∈T},

*M*, *S*, and *L* refer to the number of elements of ***x***, ***g***, and ***b***. Note that all elements of a vector which are shown in bold are non-negative real numbers. According to Cuesta et al. [34], HDF based on environmental technology D(x,g,b): ${\mathbb{R}}_{+}^{M}\times {\mathbb{R}}_{+}^{S}\times {\mathbb{R}}_{+}^{L}\to {\mathbb{R}}_{+}$ can be set up as follows:

(2) $$D\left(x,g,b\right)=\inf\left\{\theta >0|\left(x,\theta^{-1}g,\theta b\right)\in T\right\},$$

Taking DMU° as the decision making unit (DMU) under evaluation, D(x,g,b) is HDF technically efficient if and only if D=1, while D∈(0,1) indicates its state of technical inefficiency which means increasing desirable output and reducing undesired output needed to improve efficiency. Having said that, D cannot guarantee full Pareto–Koopmans efficiency because it is radially measured.

Traditional DDF measures the efficiency with the ray from the evaluated point to the projection point of the technological frontier, while HDF determines the projection point and measure efficiency along the hyperbolic path. There is also a difference between the two in the treatment of undesired outputs: Chung et al. [5] proposed that the undesired outputs should meet the joint weak disposability constraints of the output combination in DDF, which indicates the reduction of undesired output must be at the cost of a reduction in desirable output, expressed as (g,b)∈P(x)⟹(φg,φb)∈P(x),φ∈[0,1], and it require an equal sign constraint on the undesired output in the optimal programming. The HDF model deals with the desirable output and the undesired output asymmetrically, which means that under the given input conditions, the HDF allows the output set to contain fewer desirable outputs and more undesired outputs, that is, $\left(g,b\right)\in P\left(x\right)⟹\left(\varphi^{-1}g,\varphi b\right)\in P\left(x\right),\varphi \geq 1$.

If the production set *P*(*x*) satisfies the usual axioms, D(x,g,b) has the following properties:

1. Approximate homogeneity: $D\left(x,\mu^{-1}g,\mu b\right)=\mu D\left(x,g,b\right),\mu >0$;
2. Non-decreasing for weakly disposable desirable output: D(x,μg,b)≤D(x,g,b),μ∈[0,1];
3. Non-increasing for weakly disposable undesired outputs: D(x,g,μb)≤D(x,g,b),μ≥1;
4. Non-increasing for strong disposable inputs: D(x°,g,b)≤D(x,g,b),x°≥x.

### 2.2. HDF for Energy Environmental Efficiency

In order to measure the efficiency including the energy factor, the energy input index $e\in {\mathbb{R}}_{+}$ is introduced. The following technology sets *V* can be defined by

(3) V={(x,e,g,b)|(x,e) can produce (g,b),x≥0,e≥0,g≥0,b≥0},

When measuring the energy and environmental efficiency, the reducibility of other non-energy inputs should not be taken into account [35]. The *EEE* should be measured from three perspectives: reduction of energy input and undesirable output and expansion of desirable output. If non-energy input ***x*** is set as strong disposable, energy input ***e*** is set as weak disposable, and other indicators remain unchanged, then the *EEE* can be defined by HDF $D\left(x,e,g,b\right):{\mathbb{R}}_{+}^{M}\times {\mathbb{R}}_{+}\times {\mathbb{R}}_{+}^{S}\times {\mathbb{R}}_{+}^{L}\to {\mathbb{R}}_{+}$, which can be expressed as:

(4) $$EEE=D\left(x,e,g,b\right)=\inf\left\{\theta >0|\left(x,\theta e,\theta^{-1}g,\theta b\right)\in V\right\},$$

For DMU° which is under evaluation with the input-output vector $z^{o}=\left(x^{o},e^{o},g^{o},b^{o}\right)$, the non-parametric DEA representation under variable return to scale (VRS)-based technology of *EEE* can be represented as follows in optimal programming:

(5) $$\begin{array}{lll} EEE=\theta^{*}= & \min & \theta \\ & s.t. & \underset{j\in J}{\sum }\lambda_{j}x_{j}\leq x^{o},\\ & & \underset{j\in J}{\sum }\lambda_{j}e_{j}\leq \theta e^{o},\\ & & \underset{j\in J}{\sum }\lambda_{j}g_{j}\geq \theta^{-1}g^{o},\\ & & \underset{j\in J}{\sum }\lambda_{j}b_{j}\leq \theta b^{o},\\ & & \underset{j\in J}{\sum }\lambda_{j}=1,\lambda_{j}\geq 0,j\in J \end{array}$$

where $\lambda_{j}$ is the strength variable for constructing the convex combination of input-output indicators, and j∈J={1,2,3,⋯,n} is the ordinal number of DMUs. The model (5) measures the efficiency under the assumption of constant return on scale (CRS) when the convex combination constraint $\underset{j\in J}{\sum }\lambda_{j}=1$ is removed.

Note that the DEA model for measuring HDF distance is a non-linear programming, especially difficult to solve under the VRS assumption, and with no way to obtain the scale effect. Fare et al. proposed a non-parametric method of linear approximation to estimate HDF efficiency [18], but Zofio and Lovell [36] pointed out that the error of the approximation result will increase with the distance of the measured point from the technological frontier. That is to say, the lower the efficiency, the less reliable its approximate result. Fare et al. [23] further proposed an iterative method based on linear approximation, using the approximate relationship between the DDF model and the HDF model, combining the characteristics of the dual model and the original constraints to perform iterative calculations, to control the error accuracy to obtain the final efficiency. Yet they only discuss the case where all indicators were weakly disposable, with no thought for uncontrollable variables and undesirable outputs. Following their idea, we will improve the approach to apply to the approximation of *EEE* within the non-parametric framework of DEA.

### 2.3. An Improved Non-Parameters Approach for Estimating HDF

Model (5) can be expressed in a compact form as

(6) $$\begin{array}{ccc} EEE=\theta^{*}= & \min & \theta \\ & s.t. & z_{h}^{o}\left(\theta \right)=\left(x^{o},\theta e^{o},\theta^{-1}g^{o},\theta b^{o}\right)\in V, \end{array}$$

where $z_{h}^{o}\left(\theta \right)$ represents the hyperbolic path with parameter *θ* passing through DMU°, and $\theta^{*}$ refers to the optimal solution of the model. Given θ∈(0,1], it is well known that $\beta \to 0\Rightarrow \theta =e^{-\beta }\approx 1-\beta$ by applying Taylor’s theorem. Accordingly, model (6) can be approximated using the following model

(7) $$\begin{array}{cc} EEE^{o}=\theta^{*}\approx & e^{-\beta^{*}}\\ \beta^{*}=\max & \beta \\ s.t. & z_{d}^{o}\left(\beta \right)=\left(x^{o},\left(1-\beta \right)e^{o},\left(1+\beta \right)g^{o},\left(1-\beta \right)b^{o}\right)\in V, \end{array}$$

where $z_{d}^{o}\left(\beta \right)$ represents the straight path with parameter β passing through DMU°, and $\beta^{*}$ refers to the optimal solution inside. In order to estimate $\beta^{*}$, we can utilize standard linear programming to rewrite the optimal model as follows:

(8) $$\begin{array}{lll} \beta^{*} & =\max & \beta \\ & s.t. & \underset{j\in J}{\sum }\lambda_{j}x_{j}+\beta d_{x}\leq x^{o},\\ & & \underset{j\in J}{\sum }\lambda_{j}e_{j}+\beta d_{e}\leq e^{o},\\ & & -\left(\underset{j\in J}{\sum }\lambda_{j}g_{j}-\beta d_{g}\right)\leq -g^{o},\\ & & \underset{j\in J}{\sum }\lambda_{j}b_{j}+\beta d_{b}\leq b^{o},\\ & & \underset{j\in J}{\sum }\lambda_{j}=1,\lambda_{j}\geq 0,j\in J, \end{array}$$

where $d_{x}=0,d_{e}=e^{o},d_{g}=g^{o},d_{b}=b^{o}$.

Apparently this model (8) actually evaluates the DDF of DMU° with the particular directional vector $d=\left(-d_{x},-d_{e},d_{g},-d_{b}\right)=\left(0,-e^{o},g^{o},-b^{o}\right)$. That is to say, HDF model is a special type of DDF model in a manner. With the help of relation between DDF and HDF, *EEE* the optimal solution based on HDF can be approximated by solving the DDF model. Yet the farther $\beta^{*}$ is to 0, the less accurate the approximated EEE. In the light of Fare et al., an linear programming (LP)-based computational algorithm needs to be applied to obtain the exact value of EEE.

The model (8) can be transformed into the following dual model:

(9) $$\begin{array}{cc} \min & q^{\prime }x^{o}+p^{\prime }e^{o}+v^{\prime }b^{o}-w^{\prime }g^{o}+u\\ s.t. & q^{\prime }x_{j}+p^{\prime }e_{j}+v^{\prime }b_{j}-w^{\prime }g_{j}+u\geq 0\\ & p^{\prime }e^{o}+v^{\prime }b^{o}+w^{\prime }g^{o}=1\\ & j\in J, \end{array}$$

where $q^{\prime },p^{\prime },v^{\prime },w^{\prime }$ refer to the transpose vectors of the shadow prices of x,e,b,g in turn, of which optimal values determine the gradient parameters of the supporting hyperplane of the technology set *V*. u is a variable corresponding to convexity constraints, and its optimal value determines the type and size of returns to scale. By solving model (8) and model (9), a pair of dual solutions $\left(\lambda^{1},\beta^{1}\right)$ and $\left(q^{1},p^{1},v^{1},w^{1},u^{1}\right)$ can be obtained. Then ***H*^1^**: $q^{1\prime }x+p^{1\prime }e+v^{1\prime }b-w^{1\prime }g+u=0$ is a supporting hyperplane for *V* at the projection point $z_{d}^{o}\left(\beta^{1}\right)=\left(x^{o},\left(1-\beta^{1}\right)e^{o},\left(1+\beta^{1}\right)g^{o},\left(1-\beta^{1}\right)b^{o}\right)$; thereby, the equation for solving the intersection $z_{h}^{o}\left(\theta^{1}\right)$ of the hyperbolic curve $z_{h}^{o}\left(\theta \right)=\left(x^{o},\theta e^{o},\theta^{-1}g^{o},\theta b^{o}\right)$ with hyperplane ***H*^1^** can be expressed as follows:

(10) $$q^{1\prime }x^{o}+\theta^{1}p^{1\prime }e^{o}+\theta^{1}v^{1\prime }b^{o}-\frac{1}{\theta^{1}}w^{1\prime }g^{o}+u=0$$

Let $a=p^{1\prime }e^{o}+v^{1\prime }b^{o},b=q^{1\prime }x^{o}+u,c=-w^{1\prime }g^{o}$, Equation (10) can be rewritten by:

(11) $$a\theta^{1}+b+\frac{c}{\theta^{1}}=0$$

The solutions of Equation (11) are as follows:

$$\begin{array}{cccc} \mathrm{if} & a\neq 0, & c=0 & \theta^{1}=-b/a\\ \mathrm{if} & a=0, & c\neq 0 & \theta^{1}=-c/b\\ \mathrm{if} & a\neq 0, & c\neq 0 & \theta^{1}=\frac{-b+\sqrt{b^{2}-4ac}}{2a} \end{array}$$

The next step is to test whether $\theta^{1}$ can meet the constraint *Z*^1^: $z_{h}^{o}\left(\theta^{1}\right)=\left(x^{o},\theta^{1}e^{o},\frac{1}{\theta^{1}}g^{o},\theta^{1}b^{o}\right)\in V$. Our approach is to start by constructing a non-parametric model for measuring DDF, as shown in model (12), where the original reference set $\left\{DMU_{j}|j\in J\right\}$ is used as the reference set, $\left(x^{*},e^{*},g^{*},b^{*}\right)$ represents the input-output vector of the unit $DMU^{*}$ evaluated, and the direction vector of DDF is $\left(-x^{*},-e^{*},g^{*},-b^{*}\right)$.

(12) $$\begin{array}{lll} \gamma^{*} & =\max & \gamma \\ & s.t. & \underset{j\in J}{\sum }\lambda_{j}x_{j}+\gamma x^{*}\leq x^{*},\\ & & \underset{j\in J}{\sum }\lambda_{j}e_{j}+\gamma e^{*}\leq e^{*},\\ & & -\left(\underset{j\in J}{\sum }\lambda_{j}g_{j}-\gamma g^{*}\right)\leq -g^{*},\\ & & \underset{j\in J}{\sum }\lambda_{j}b_{j}+\gamma b^{*}\leq b^{*},\\ & & \underset{j\in J}{\sum }\lambda_{j}=1,\lambda_{j}\geq 0,j\in J \end{array}$$

The point $z_{h}^{o}\left(\theta^{1}\right)$ is taken as the given input-output vector of the virtual $DMU^{*}$ to find the optimal solution $\gamma^{*}$ of model (12). If γ∗ = 0, it means that the point $z_{h}^{o}\left(\theta^{1}\right)$ is on the envelope of the technological frontier of *V*, and $\theta^{1}$ satisfies the constraint condition *Z*^1^, that is, $\theta^{1}$ is the final exact approximation of *EEE*. If $\gamma^{*}\neq 0$, it indicates that $\theta^{1}$ does not satisfy the constraint condition Z^1^, and $z_{h}^{o}\left(\theta^{1}\right)$ is outside the technology set, which means the following iteration algorithm needs to be implemented:

1. Reconstruct a new direction vector $d^{1}$ from DMU° to point $z_{h}^{o}\left(\theta^{1}\right)$: $d^{1}=\left(-d_{x}^{1},-d_{e}^{1},d_{g}^{1},-d_{b}^{1}\right)=\left(-\left(x^{o}-x^{o}\right),-\left(\theta^{1}e^{o}-e^{o}\right),\frac{1}{\theta^{1}}g^{o}-g^{o},-\left(\theta^{1}b^{o}-b^{o}\right)\right)$;
2. Solving the DMU° for the DDF model (8) and its dual model (9) with ***d*1** as the direction vector, we can get the second pair of dual solutions $\left(\lambda^{2},\beta^{2}\right)$ and $\left(q^{2},p^{2},v^{2},w^{2},u^{2}\right)$, and then obtain another supporting hyperplane equation **H^2^**: $q^{2\prime }x+p^{2\prime }e+v^{2\prime }b-w^{2\prime }g+u=0$ for V;
3. Let the intersection of **H^2^** and hyperbolic curve $z_{h}^{o}\left(\theta \right)=\left(x^{o},\theta e^{o},\theta^{-1}g^{o},\theta b^{o}\right)$ named $z_{h}^{o}\left(\theta^{2}\right)$, which satisfies the equation of the hyperplane **H^2^**, so we can get a model with the parameters $\left(q^{2},p^{2},v^{2},w^{2},u^{2}\right)$ and the unknown factor $\theta^{2}$ as the model (10). Then we solve the equation to obtain the value of $\theta^{2}$;
4. Constructing a new constraint test condition *Z*^2^: $z_{h}^{o}\left(\theta^{2}\right)=\left(x^{o},\theta^{2}e^{o},\frac{1}{\theta^{2}}g^{o},\theta^{2}b^{o}\right)\in V$, which needs to test whether $\theta^{2}$ is the optimal HDF solution. If true, the iteration stops, otherwise it continues to reconstruct a new directional vector $d^{2}$ from DMU° to point $z_{h}^{o}\left(\theta^{2}\right)$, and returns to step 1.

### 2.4. Efficiency and Malmquist Index within Global Reference

Traditional DEA efficiency is evaluated by taking the DMU set of each year as the reference set, and there is a different technology frontier or reference standard for each year. Therefore, comparison of the efficiencies of the same DMU in different years can only reflect the relative distance change between the DMU and the technology frontiers of each year and cannot embody the absolute disparity of efficiency from year to year. In this paper, with reference to Pastor and Lovell [37], the global energy and environmental efficiency (GEEE) is calculated, and the global Malmquist index is further calculated by GEEE to measure the change of energy and environmental productivity (EEP) under the framework of total factors.

To calculate GEEE, we only need to define the DMU sequence number set J in the DEA model as J={1,2,3,⋯,NT}, where *T* is the number of periods of panel data and N is the number of DMUs per year. Unlike the geometric mean form of traditional Malmquist index, global Malmquist index takes the common frontier consisting of all DMUs each year as the reference set, and due to a single index, it satisfies the transitivity and can be multiplied cumulatively. The energy and environmental productivity changes (EEPC) from year t to year *t* + 1 can be expressed as follows:

(13) $$EEPC=EEP^{t,t+1}=\frac{GEEE^{t+1}}{GEEE^{t}}=\frac{EEE_{g}^{t+1}\left(x^{t+1},e^{t+1},g^{t+1},b^{t+1}\right)}{EEE_{g}^{t}\left(x^{t},e^{t},g^{t},b^{t}\right)},$$

where $EEE_{g}^{t}$ represents the energy and environmental efficiency of period *t* under the global reference. $EEP^{t,t+1}=1+\alpha /100$ indicates that EEP has increased by *α*% from *t* to *t* + 1 year, and also reflects absolutely the change of GEEE. In order to explore the internal causes of the change in EEP, EEPC under the CRS assumption can be decomposed according to Fare et al. [38] as follows:

(14) EEPC(CRS)=EC(CRS)∗TC(CRS)=PEC(VRS)∗SEEC∗TC(CRS),

$EC\left(CRS\right)=\frac{EEE^{t+1}\left(x^{t+1},e^{t+1},g^{t+1},b^{t+1};CRS\right)}{EEE^{t}\left(x^{t},e^{t},g^{t},b^{t};CRS\right)}$ refers to the relative change of the technical efficiency within different frontiers each year, which reflects the changes in current technical application ability, resource organization, and management capabilities at the current scale.

$TC\left(CRS\right)=\frac{EEE_{g}^{t+1}\left(x^{t+1},e^{t+1},g^{t+1},b^{t+1};CRS\right)/EEE^{t+1}\left(x^{t+1},e^{t+1},g^{t+1},b^{t+1};CRS\right)}{EEE_{g}^{t}\left(x^{t},e^{t},g^{t},b^{t};CRS\right)/EEE^{t}\left(x^{t},e^{t},g^{t},b^{t};CRS\right)}$ represents the relative changes in the respective technological frontiers from year *t* to *t* + 1. It should be noted that the changes in technological frontiers here are measuring the change of the projection point of the DMU on the front surface, not the change of the overall front surface. Technological efficiency change (EC) (CRS) includes both changes in the practical capabilities of the current technology and changes in efficiency caused by changes in returns under scale changes. Therefore, EC (CRS) can be decomposed into two factors as follows:

$PEC\left(VRS\right)=\frac{EEE^{t+1}\left(x^{t+1},e^{t+1},g^{t+1},b^{t+1};VRS\right)}{EEE^{t}\left(x^{t},e^{t},g^{t},b^{t};VRS\right)}$ refers to the pure technical efficiency after removing the scale effect, and $SEEC=\frac{EEE^{t+1}\left(x^{t+1},e^{t+1},g^{t+1},b^{t+1};CRS\right)/EEE^{t+1}\left(x^{t+1},e^{t+1},g^{t+1},b^{t+1};VRS\right)}{EEE^{t}\left(x^{t},e^{t},g^{t},b^{t};CRS\right)/EEE^{t}\left(x^{t},e^{t},g^{t},b^{t};VRS\right)}$ represents the relative change in technical efficiency caused by scale factors.

## 3. Material

### 3.1. Subject Investigated

In 2013, the State Council of China issued the “National Resource-based City Sustainable Development Plan” (hereinafter referred to as the “2013 Plan”), which lists the 262 resource-based cities, and only distinguishes between mining and forestry, including 126 prefecture-level cities. This paper focuses on the cities based on exhaustible resources, selecting non-forest prefecture-level cities from the list, excluding the two special resource cities of Zigong (Salt) and Jingdezhen (Ceramics) and another city Bijie (promoted to a prefecture-level city in 2011); then 107 resource-based cities are identified as the subject investigated.

### 3.2. Data Sources and Description

In order to evaluate the GEEE and energy environmental productivity change (EEPC), we took capital stock and labor as non-energy input ***x***, energy consumption as energy input ***e***, actual regional GDP as desirable output ***g*** [16,39], and three waste emissions as undesired output ***b***. The selection and processing of input-output indicators are as follows:

Capital stock (*k*). The capital stock of prefecture-level cities is estimated by using the perpetual inventory method. The basic formula is $k_{t}=\frac{I_{t}}{p_{t}}+\left(1-\delta \right)k_{t-1}$, where $I_{t}$ and $p_{t}$ are the nominal value of fixed investment and investment price index in the year *t*, and *δ* is the depreciation rate. Due to the lack of prefecture-level investment price index, we used the fixed asset investment price index of the province where the prefecture-level city is located as a substitute, and *δ* is defined by 5%. To calculate $k_{t}$, the capital stock in the base year ($k_{0})$ also needs to be evaluated, and it can be calculated by the formula $k_{0}=\frac{I_{0}}{\left(g+\delta \right)}$, where $I_{0}$ refers to the investment in the base year and *g* is the average growth of real fixed asset investment from 2003 to 2016.

Labor input (*l*). We used the total number of employees at the end of the year, that is, the total number of employees in the unit of the city and the total number of private and individual employees in the city as the total labor input of the prefecture-level city.

Energy consumption (*e*). There is a high correlation between power consumption and energy consumption, and power consumption can be used as an alternative to regional energy consumption. Due to the limited data of the consumption of fossil energy such as coal and oil in all prefecture-level cities, the power consumption of prefecture-level urban areas is used as the approximate replacement of the city’s energy consumption. Unit: kilowatt-hour

Gross regional product (*g*). We took 2003 as the base year to carry out price adjustment and calculate the actual GDP of prefecture-level cities each year as the desirable output.

In terms of three wastes, the cities’ industrial sulfur dioxide emissions (s), wastewater emissions (w), and soot emissions (d) are selected as the measures of undesirable output with equal weight.

All data mainly comes from China Urban Statistical Yearbook, Easy Professional Superior (EPS) database, and Statistical Bulletin of National Economic and Social Development of cities. In addition, the data of abnormal changes between years and changes in some urban divisions during the investigation period are modified to ensure the reliability of the data. In this paper, R language is used to program the HDF iterative model mentioned above to evaluate GEEE and EEPC as well as the relevant decomposition results. Descriptive statistics of input-output variables from 2003–2018 are shown in Table 1.

## 4. Empirical Study

### 4.1. Dividing Resource-Based Cites by RD

In this paper, RD is regarded as the dependence of a city’s economy on the exact local resources, or for example, the mining industry. To test how RD can affect the energy environmental performance, we classify these cities by RD degree to investigate the differences of GEEE and EEPC. It is typical to use the proportion of mining industry in an economy as a measure of RD in empirical analysis. However, it is difficult to find a suitable boundary in the actual division simply by the absolute measurement of the proportion of mining, in which the important influence of manufacture processing industry on economy is also ignored. Therefore, we developed a new relative indicator, the city function advantage index (FAI), to represent RD in this section.

#### 4.1.1. Using FAI to Represent RD

Above all, we need to explain the relationship between RD and city function advantage so that the FAI can be used to represent RD. For resource-based cities, usually resource mining and manufacturing processing are often the two most prominent functional departments of the basic economic activities. From the perspective of the evolution law of the function development, most of the mining regions mainly focus on the mining industry in the early stage, and gradually transition to the manufacturing industry after the mining-processing coexist stage.

**Figure 1:**
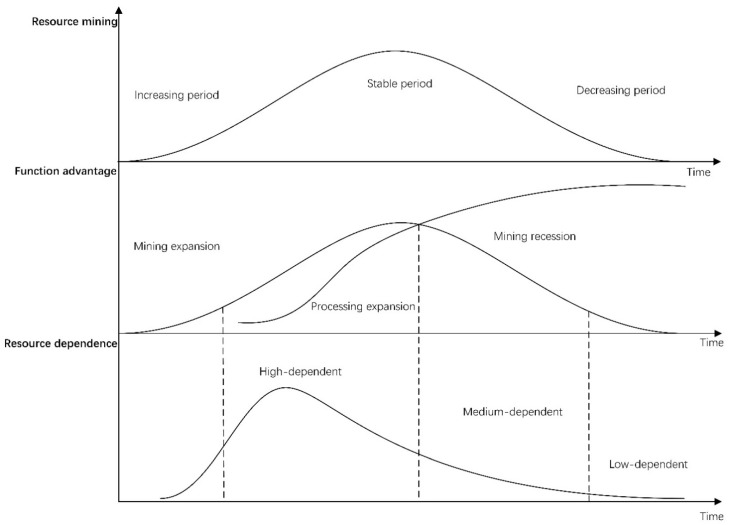
The general evolution process of the relationship between resource dependence (RD) and city function advantage.

The basic law of development and city function advantage evolution of resource-based cities is shown in Figure 1. Conveniently the resource-based city analyzed is assumed to be an agricultural economy in the early stage of development. Due to the exhaustibility of mineral resources, the resource output of a city inevitably goes through the process of increasing to stable production and then to decreasing, and accordingly, the proportion of the mining industry in the sectors of the economy also first increases and then decreases. Then the development of a city can be divided into three stages: (1) In the early stage of increasing production in a resource-based city, the prominent function is mining, and the manufacturing industry is relatively weak. Such city economic activities are extremely highly dependent on local resources, which belong to the “high-dependent” stage. (2) With the expansion of the mining industry, lower resource cost and specialized division of labor demand, the resource processing industry, upstream and downstream of the industrial chain and other related systems, is stimulated rapidly. When the economic volume of manufacturing industry begins to exceed that of mining industry, the city function changes from a single mining industry to a diversified industrial function, and the city begins to obtain the ability to utilize local resources. Economic activities tend to be decoupled from local resources, which belong to the “medium-dependent” stage. (3) When the period of resource production reduction comes, the city’s mining activity gradually declines, the function of resource output is weakened, and local resources cannot meet the demand of resource processing industry. The city begins to seek out the required resources, and other manufacturing industries are also growing because of the effect of industrial agglomeration. The comprehensive manufacturing industry begins to occupy an absolute dominant position. The relationship between such city activities and local resources is very weak, which belongs to the “low-dependent” stage. Therefore, the city function advantage evolution of resource-based cities also corresponds to the change of the relationship between economic activities and local resources, from high-dependent to low-dependent.

#### 4.1.2. Classification by RD and Region

We use the ratio of employees in mining industry and manufacturing industry to calculate FAI. With the help of the average of FAI ($\bar{FAI}$) from 2003 to 2016, we can define the RD degree of cities, in which resource-based cities can be divided into three categories: high-dependent ($\bar{FAI}$ ≥ 1), medium-dependent ($\bar{FAI_{n}}$ ≤ $\bar{FAI}$ < 1, $\bar{FAI_{n}}$ refers to the national average), and low-dependent ($\bar{FAI}$ < $\bar{FAI_{n}}$). In addition, the investigated resource-based cities are also classified by region such as east, northeast, middle, and west to discuss the difference of GEEE and EEPC, as shown in Table 2.

From the perspective of RD, high-dependent cities are mainly cities that exploit coal, oil, and other resources, with obvious characteristics of energy base, such as Yulin, Ordos, and Karamay. Medium-dependent cities are mainly heavy industrial cities that process local resources such as coal, electricity, steel, nonferrous metals, etc., maintaining a certain proportion of mining departments to support the development of resource processing industry, such as Panzhihua, Tangshan, and Huangshi. Low-dependent cities are mainly non-ferrous metal or comprehensive industrial cities, and the relative strength of mining manufacturing industry is even lower than the national level, which indicates that the resource extraction sector does not occupy the main position in the local economy, and city development relies less on local resources. Among them, there are the resource deep-processing cities such as Jinchang, Tongling, and Shizuishan, and other comprehensive industrial cities such as Huzhou, Luzhou, and Luoyang. It can be seen that the classification reflects the reality to a large extent.

### 4.2. GEEE Analysis

#### 4.2.1. Difference by RD

What are the differences of energy and environmental efficiency of resource-based cities under different degrees of RD? In the long run, it is a reasonable assumption that the scale return is constant from the macro perspective. Therefore, we used GEEE with CRS assumption, to analyze the current situation of energy and environmental efficiency from the perspectives of distribution and trend.

**Figure 2:**
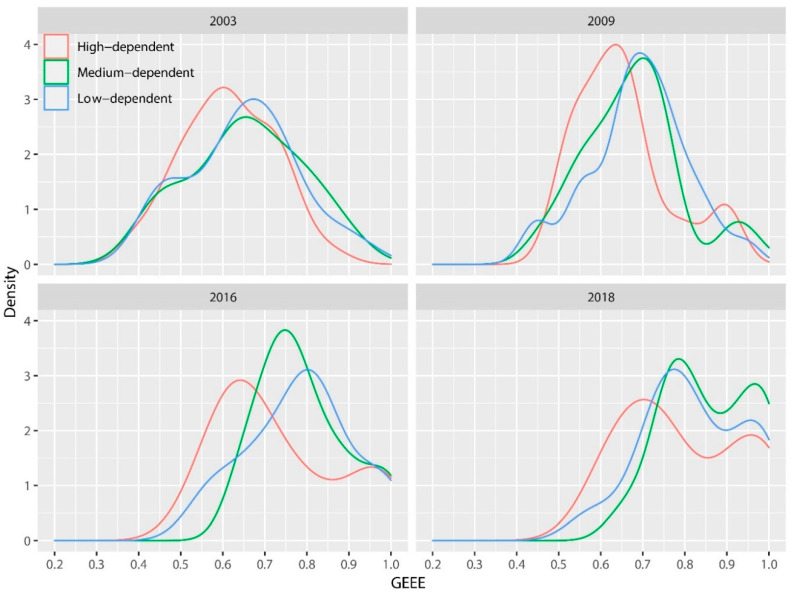
Density distribution of global energy and environmental efficiency (GEEE) for classification by RD.

Figure 2 shows the nuclear density distribution of energy and environmental efficiency of resource-based cities in 2003, 2009, 2016, and 2018, from which we can get the following four basic judgments: (1) The wave peaks of three types of efficiency distribution move to the right as time goes on, and the right shift range from 2009 to 2016 is more obvious, which shows that GEEE of resource-based cities in 2003–2018 is constantly improving, especially during the “new normal” of China’s economy. Driven by the structural reform of the supply-side, the work of energy conservation and environmental protection has achieved remarkable results. (2) There are differences in the distribution of different types of cities, and the right shift of high-dependent cities is significantly smaller than that of other two types. The work by Huang et al. [25] also shows that the ecological efficiency of the resource-development group is significantly lower than that of resource-utilization areas, which shows that the efficiency of strong-dependence cities has improved more slowly than the other two types of cities in recent years. The reason may be that due to the rich local resources and low energy cost, the strong-dependence cities have caused energy waste, curbed their investment in research and development of local energy conservation and environmental protection technologies, and hindered the introduction and application of relevant new technologies. (3) The efficiency alienation within strong-dependence cities is apparent, while the other two tend to be relatively concentrated. The wave peak of strong-dependence cities decreases year by year, and the distribution tends to be gentle gradually, which indicates that the efficiency of each strong-dependence city is gradually differentiated, and the efficiency improvement from year to year is not synchronous, while the efficiency improvement of the other two types shows the same convergence phenomenon. (4) The distribution and evolution characteristics of GEEE of medium-dependence and weak-dependence cities are basically consistent and synchronous, which indicate that there may not be apparent boundaries and differences between these two types of cities in energy structure optimization, energy and environmental technology research and development, and application.

**Figure 3:**
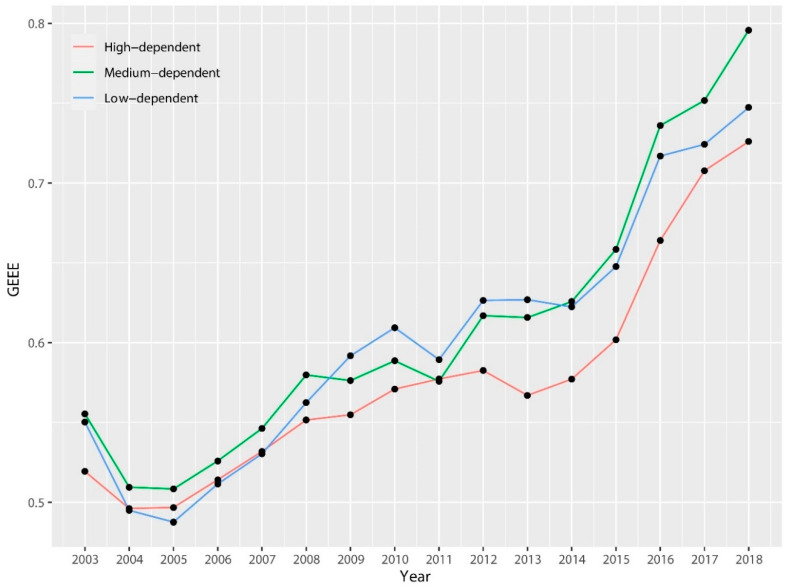
Trend of GEEE for classification by RD from 2003 to 2018.

Figure 3 depicts the average trend of GEEE in three types of cities. During the sample period, all kinds of efficiency almost doubled, and the average trend of medium-dependence and weak-dependence cities basically coincide; but the efficiency of strong-dependence city is always the lowest, and the efficiency gap from the other two is gradually expanding, which further confirms the previous analysis. From the perspective of the trend structure of efficiency improvement, the three types of cities have roughly gone through three stages: rising (2003–2009), platform (2009–2014), and recovery rising (2014–2018). These stages are just in line with the real economic cycle: the global economic crisis caused by the U.S. subprime mortgage crisis in 2009, resulting in a sharp fall in global resource prices and a severe setback in resource-related industries, which caused a fatal blow to the development of resource-based cities, and the GEEE is basically in the stage of platform consolidation in the next five years with slow improvement. In 2013, the Chinese government issued “2013Plan” to supply specific classification guidance for sustainable development of resource-based cities, and then in 2014 the structural reform of supply-side was implemented with the goal of optimizing economic structure, improving total factor productivity, and improving economic quality. Due to the incentive of “three removal, one reduction and one compensation”, backward production capacity was further eliminated, and the resource industry has been rapidly restored, meanwhile GEEE of resource-based cities has also been greatly improved, especially in 2014–2018.

#### 4.2.2. Difference by Region

**Figure 4:**
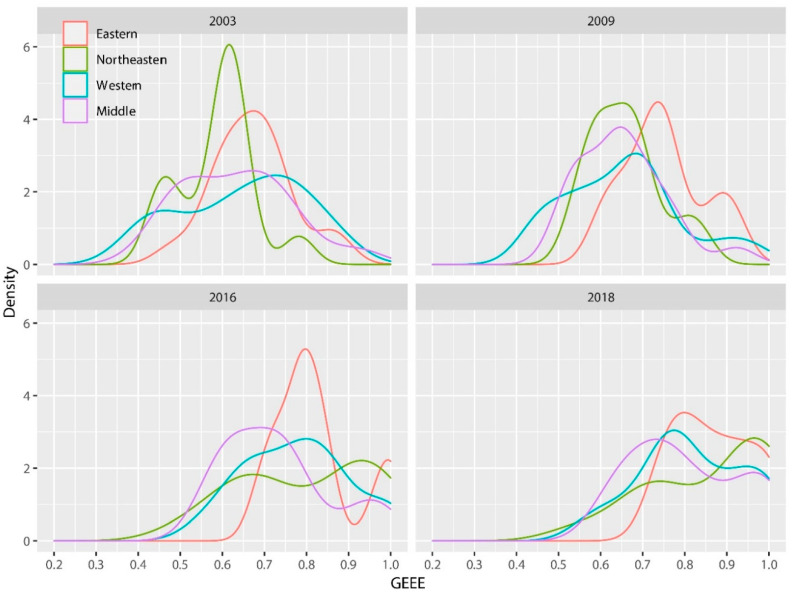
Density distribution of GEEE for classification by region.

Figure 4 shows that the right-shift of the efficiency distribution in the east and northeast during the sample period is more apparent than that in the middle and west, which indicates that the efficiency in the east and northeast has improved more, especially in the northeast. As the old industrial base, most of the resource-based cities in the northeast of China have basically entered the middle and late stage of resource development, and have been undergoing a critical period of economic transformation, with the strategic support of “revitalizing the old industrial base in Northeast China” of the middle government, a large amount of funds, and the policy incline to the northeast, which lead to significant improvement of GEEE in that region. In contrast, the efficiency distribution in the middle and western regions has not changed much, with a soft overall shift to the right. It may be that in Western Development the resource-based cities in the middle and west have undertaken more resource mining and processing functions for the national economic development, and gradually replaced the northeast as a new energy and raw material base. An irresistible temptation of the short-term achievement brings about the lock of reasonable development planning and effective supervision over resources of the local government in the middle and west, resulting in resource waste and environmental damage.

**Figure 5:**
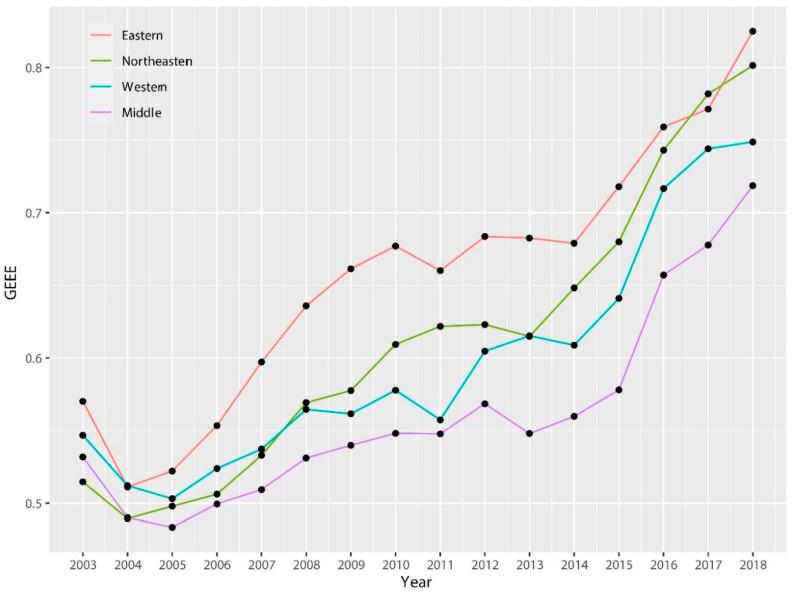
Trend of GEEE for classification by region from 2003 to 2018.

Figure 5 shows that the average GEEE of the east is always higher than other regions, and the northeast is always catching up, with its average doubled during the sample period. In comparison, the middle and west have been increasing slowly, especially for resource-based cities in the middle region. GEEE of the middle region was comparable to the east in 2003, but since 2012, the efficiency has lagged behind other regions, and the gap with other regions has gradually widened, showing a phenomenon of “middle collapse”. The possible reasons for the “middle collapse” may be as follows: (1) Regional development in the middle is confronted with double squeeze from the west and east, is not rich in resources like the west, and has a lower technological level and value of the resource industry chain as in the east. (2) Compared with other national regional coordination strategy such as Western Development and Northeast Revitalization, the starting point for the implementation of the Middle Rising is relatively late, with insufficient related policy support. (3) As domestic environmental problems become more serious, environmental regulations in the middle are also becoming increasingly strict along with the east, which has led to a slowdown in the rate of capital accumulation, and retarded the process of technological innovation and improvement of GEEE. In addition, from the perspective of the trend structure, GEEE by region also shows three-stage law, among which the platform adjustment period for the efficiency of the middle region is also more obvious and suffered more serious external shocks.

### 4.3. EEPC Analysis

#### 4.3.1. Overall EEPC

By calculating EEPC and its decomposition of each city, we define the geometric mean of that as the average level of overall resource-based cities. The cumulative trend of EEPC and its decomposition for resource-based cities from 2003 to 2016 is shown in Table 3, with the static value, such as EEP, of each item for the base year 2003 defined by 1.

The overall energy environmental productivity (EEP) is generally on the rise, with an average annual growth rate of 2.6% during the sample period. From the perspective of EEPC’s decomposition, technological change (TC) has always been the most important factor in improving overall EEP, with an average annual growth rate of 2.9%, which is even higher than the latter. The relative technical efficiency, measured by EC, has remained at a relatively low level, indicating that changes in technological efficiency have little effect on the improvement of energy and environmental productivity. Furthermore, technical efficiency has been in a downward trend since 2012, resulting in an average annual decrease of 0.3% of relative technical efficiency, which exerts a negative impact on the improvement of EEP.

By further separating the scale effect on efficiency change (SEEC) by the scale factor of EC, the average annual change in pure technical efficiency (PEC) is −0.2%, indicating that the decline in PEC is the main reason for the decline in technological efficiency. Although with the rapid development of industrial Internet, clean energy, and environmental protection industry, the overall urban energy and environmental technology have greatly improved during these years. The resource-based cities have relatively retrogressed in terms of technology utilization, resource allocation, and environmental management on the existing scale, that is, there is still much room for improvement. In addition, inefficient scale is another important factor that causes the technological efficiency to decline. The average annual change in the SEC is −0.1%. This reveals that inspired by the prosperity of the previous resource market, local government in resource-based cities tend to lack short-term and reasonable planning to pursue short-term benefits, blindly expand the industrial scale, and ignore the transformation and upgrade of industrial energy-saving and environmental protection technologies. It leads to these cities’ excess low-end capacity and insufficient high-end capacity in a slump of resource market after 2012, falling into a weird, large-scale but inefficient situation, which is also in line with the “three go, one drop, one supplement” policy implemented by the middle government of this period.

There is not much research on the energy and environmental productivity of resource-based cities, but in the study of energy efficiency in China, changes in technological efficiency are considered to be the main reason for the increase in energy productivity, followed by technological progress. The conclusions are similar to the provincial-level research conclusions of Zhang and Jiang [40], and we take the view that technological progress is the main factor for improving energy and environmental productivity. Further research found that the relative technological efficiency of resource-based cities even deteriorated; far from keeping up with the pace of technological progress, and ineffective scale expansion, low technological application levels and resource and environmental management capabilities are the main reasons for relative technological efficiency regressions.

#### 4.3.2. EEPC for Classification

The cumulative value of energy and environmental productivity of resource-based cities and related decomposition in classification by RD and region are shown in Table 4 and Table 5. From the view of resource-dependent classification, the medium-dependent group has the fastest growth in energy and environmental productivity. The high-dependent group is slightly lower than the other two categories most of the time, and its cumulative EEPC catches up with the low-dependent cities only at the end of the sample period. From the perspective of region classification, the order of the growth rate of energy and environmental productivity from high to low is northeast, east, west, and middle, indicating that environmental protection in the northeast has achieved remarkable results. The middle region has the worst performance, and the gap with other regions has expanded. The total cumulative growth rate of the middle is only 35.1%.

For the decomposition result of EEPC, from the perspective of resource-dependent classification, the highest annual growth rate of energy and environmental productivity in high-dependent cities is 2.26%, and that of the other two types is 2.06% and 2.43%. In terms of the contribution of decomposition factors, the technological progress of each group has generally exceeded 100%, which is the most significant factor for the growth of energy and environmental productivity. The high-dependent group has reached 121.31%, the highest in technological progress, but the scale factors of pure technological efficiency change and technological change are −20.74% and −14.19%, respectively the lowest. This again shows that the biggest problem of improving energy and environmental productivity of high-dependent cities lies in the insufficient application of source environment technology, the invalid allocation of resources, and the excessive production scale. From the perspective of region classification, compared with other regions, the contribution of technological change to energy and environmental productivity in the west is the smallest, less than 100%, while that of pure technological efficiency is the highest, at 7.68%. This shows that resource-based cities in the west have made great progress in the application of cutting-edge technology. The contribution of pure technological change in the middle is the lowest, at only −20.24%, which also leads to the low growth of total productivity. In addition, the contribution of pure technology change and scale factor change of resource-based cities in the east is also negative.

### 4.4. Classification by Combining Static Efficiency and Dynamic Productivity Changes

In order to examine the advantages and disadvantages of resource-based cities in terms of energy and environmental productivity, resource-based cities are divided from two aspects: static efficiency of energy efficiency and dynamic changes in productivity. Taking the average of the average annual growth rate of static efficiency and productivity as the boundary, 107 resource-based cities are classified into four groups of HH, HL, LH, and LL, where the first letter indicates the size of static efficiency and the second indicates the average annual growth rate of productivity. Cities in the HH group, in which energy and environmental efficiency and dynamic productivity are at a relatively high level, are the first echelon of resource-based cities. Cities in the HL group, in which static efficiency is relatively high, but the productivity growth rate is low, are facing the risk of being overtaken by other cities. For cities in the LH group, their efficiencies are low, but there is a rapid productivity growth, and there is greater room for future growth. For cities in the LL group, not only are static efficiencies low, but also the productivity growth rate is lower than the average level and the growth potential is limited; this group belongs to the end of the echelon and needs to be focused on by the government.

Table 6 lists the four groups of 107 cities. The number of cities in each group is relatively uniform. Among them, the number of cities in the HH group is relatively few, only 24, and the most cities are in the LH group, at 31. It is clear that there are obvious differences in energy and environmental productivity among cities, and the potential for energy conservation and environmental protection is huge. Therefore, the government should pay attention to the diversity of cities in energy and environmental productivity, when formulating the tasks of energy conservation and environmental protection policies and index decomposition. In order to implement policies according to the city, local governments must not only recognize the advantages of the city and to give full play to the advantages, but also find out the reality of its shortcomings and introduce appropriate policies to make up for these shortcomings. The middle government can comprehensively guide the optimization of energy structures and environmental protection policies of different types of resource-based cities from the perspective of RD and region, so as to achieve sustainable and coordinated development of various resource-based cities and promote their economic transformation and upgrading.

## 5. Conclusions and Enlightenment

Efficiency is a vital element in order to achieve the benefits of rich regional resources [41]. The hyperbolic distance function (HDF), different from other DEA models, considers both the desirable output growth and the undesirable output reduction asymmetrically. Based on the idea of Fare et al. [23], this paper proposes an LP iterative algorithm to estimate HDF, which includes undesirable output and uncontrollable index. The energy environment efficiency and productivity change and decomposition of resource-based cities are calculated under the global reference framework and are analyzed by the classification of RD and region. The main conclusions are as follows: (1) On the whole, the annual growth rate of energy and environmental productivity of resource-based cities in China is 2.6%, which is mainly due to technological changes. Work by Zhang et al. [42] also show that technical changes are the dominant factors behind productivity change for China’s cities. The decline of relative technological efficiency hinders the further growth of productivity, and scale factor is the main reason for the decline of technological efficiency. (2) For the classification of RD, the energy and environmental efficiency of high-dependent group is significantly lower than that of the medium and low group. Using province-level data, Cheng et al. [43] also found that the more abundant a province’s natural resources, the lower the green total factor productivity, similar as Wang et al. [44]. The energy and environment productivity of the medium group has the highest growth. The regression of relative pure technical efficiency and scale diseconomy hinders the growth of productivity of the high-dependent group. (3) For classification of regions, the middle and west perform worse than the east and northeast. The east has the highest energy and environment efficiency, while the northeast has the fastest growth rate of productivity. Both the static efficiency and the dynamic change in the middle region show the phenomenon of “collapse in the middle”, and the great retreat of relative pure technical efficiency is the main reason for its low productivity growth. (4) The city division based on the static efficiency and the dynamic productivity change shows that the advantages and disadvantages of different resource-based cities in terms of energy and environmental productivity are obviously different, which needs to be concerned in the process of policy-making.

Based on the above research, this paper has come to the following conclusions: (1) The empirical results strongly show that since 2013, the supply side structural reform of China with the main purpose of improving TFP has achieved remarkable results, and the energy and environmental efficiency and productivity growth rate of resource-based cities have been greatly improved. But there is still much room for improvement, consistent with the findings of Yan et al. [16]. We should continue to promote relevant reform unswervingly, but more attention needs to be paid to the practical application of technology and the insufficiency of resource allocation management efficiency. (2) Low relative technical efficiency and uneconomical scale are the main reasons for the poor performance of high-dependent cities in energy and environmental productivity, which also shows that RD may have a negative impact on energy and environmental efficiency. In addition, the problem of “middle collapse” also needs to attract the attention of governments. Similarly, Wang and Chen [45] also found that the central and western regions have a significant “resource curse” effect in the context of eco-efficiency, in which natural resource endowment significantly inhibits the growth of regional eco-efficiency, mainly manifested by natural resource endowment to human capital and other industries. (3) Combining the classification of static efficiency and dynamic change is helpful to accurately understand the current situation of energy utilization and environmental protection efficiency of each resource-based city. Based on the classification and guidance of sustainable development of resource-based cities, it is necessary to further implement city-specific policies [46].

## Figures and Tables

**Table 1:** Descriptive statistics of input-output variables from 2003–2018.

Type	Vars	Unit	N	Mean	sd	Min	Max
input	k	million yuan	1712	62,869.57	55,258.67	7967.23	245,883.17
	l	person	1712	53.71	32.20	14.58	146.67
	e	million kwh	1712	567,860.18	612,544.50	28,967.63	2,687,428.3
desirable output	g	million yuan	1712	83,888.51	81,698.09	10,762.87	365,580.88
undesirable output	s	ton	1712	60,857.38	51,269.11	3,555.75	212,108.63
	w	million tons	1712	4718.80	3867.93	397.78	16,080.38
	d	ton	1712	32,008.55	31,184.83	2070.83	141,027.43

**Table 2:** The classification of 107 resource-based cities by RD and region.

High-Dependent (37)			Medium-Dependent (41)			Low-Dependent (29)		
City	$\bar{FAI}$	Region	City	$\bar{FAI}$	Region	City	$\bar{FAI}$	Region
Qitaihe	8.529	Northeastern	Ordos	0.980	Western	Zibo	0.161	Eastern
Qingyang	8.086	Western	Zhaotong	0.984	Western	Sanming	0.166	Eastern
Hegang	4.811	Northeastern	Suzhou	0.935	Central	Lincang	0.157	Western
Yan’An	4.741	Western	Guangyuan	0.933	Western	Xianyang	0.163	Western
Huaibei	4.693	Central	Chifeng	0.875	Western	Baoshan	0.105	Western
Shuangyashan	4.489	Northeastern	Linfen	0.870	Central	Ezhou	0.131	Central
Yangquan	4.352	Central	Chenzhou	0.787	Central	Ganzhou	0.108	Central
Pingliang	3.866	Western	Tai’An	0.735	Eastern	Linyi	0.127	Eastern
Jincheng	3.852	Central	Dazhou	0.721	Western	Chizhou	0.106	Central
Huainan	3.803	Central	Xuzhou	0.683	Eastern	Shaoguan	0.114	Eastern
Jixi	3.578	Northeastern	Weinan	0.621	Western	Luyang	0.081	Central
Guangan	3.542	Western	Pingxiang	0.617	Central	Pu’Er	0.062	Western
Songyuan	3.453	Northeastern	Handan	0.620	Eastern	Chuzhou	0.072	Central
Shuozhou	3.300	Central	Baiyin	0.631	Western	Xinyu	0.076	Central
Datong	2.770	Central	Qujing	0.594	Western	Baotou	0.070	Western
Panjin	2.612	Northeastern	Yichun	0.498	Central	Yunfu	0.057	Eastern
Kalamay	2.612	Western	Fushun	0.433	Northeastern	Huzhou	0.056	Eastern
Fuxin	2.323	Northeastern	Tangshan	0.433	Eastern	Shizuishan	0.038	Western
Liupanshui	2.116	Western	Xingtai	0.419	Eastern	Baoji	0.047	Western
Liaoyuan	2.039	Northeastern	Shaoyang	0.373	Central	Hezhou	0.043	Central
Tongchuan	2.017	Western	Hechi	0.345	Central	Anshan	0.037	Northeastern
Dongying	1.708	Eastern	Jiaozuo	0.322	Central	Luzhou	0.031	Western
Yulin	1.677	Western	Baise	0.318	Central	Tongling	0.032	Central
Sanmenxia	1.656	Central	Loudi	0.316	Central	Nanping	0.029	Eastern
Puyang	1.557	Central	Zhangjiakou	0.308	Eastern	Xuancheng	0.019	Central
Daqing	1.523	Northeastern	Huangshi	0.284	Central	Nanchong	0.013	Western
Hulun Buir	1.423	Western	Longyan	0.281	Eastern	Yuncheng	0.023	Central
Jinzhong	1.421	Central	Panzhihua	0.250	Western	Suqian	0.020	Eastern
Wuhai	1.410	Western	Ma’Anshan	0.243	Central	Jinchang	0.007	Western
Xinzhou	1.391	Central	Wuwei	0.243	Western			
Zaozhuang	1.337	Eastern	Chengde	0.251	Eastern			
Longnan	1.303	Western	Anshun	0.205	Western			
Jining	1.191	Eastern	Tonghua	0.218	Northeastern			
Lvliang	1.147	Central	Zhangye	0.210	Western			
Hebi	1.149	Central	Huludao	0.219	Northeastern			
Changzhi	1.134	Central	Ya’An	0.220	Western			
Pingdingshan	1.100	Central	Laiwu	0.215	Eastern			
			Bozhou	0.211	Central			
			Hengyang	0.191	Central			
			Benxi	0.167	Northeastern			
			Nanyang	0.183	Central			

Note: data comes from China Urban Statistical Yearbook from 2003–2018.

**Table 3:** Cumulative value of EEPC and its decomposition of overall resource-based cities from 2003 to 2018.

Year	EEPC	EC	PEC	TC	SEEC
2004	0.925	1.008	1.016	0.918	0.992
2005	0.921	1.041	1.035	0.885	1.006
2006	0.957	1.016	1.017	0.942	0.998
2007	0.992	1.027	1.018	0.966	1.009
2008	1.044	1.009	1.010	1.035	0.999
2009	1.058	0.989	1.008	1.071	0.981
2010	1.086	0.973	1.003	1.117	0.970
2011	1.072	0.976	0.999	1.097	0.978
2012	1.122	1.002	1.012	1.120	0.990
2013	1.111	0.958	0.970	1.160	0.987
2014	1.123	0.930	0.954	1.207	0.975
2015	1.174	0.910	0.942	1.290	0.966
2016	1.303	0.940	0.961	1.387	0.978
2017	1.347	0.986	0.982	1.366	1.003
2018	1.400	0.966	0.973	1.449	0.993
Average growth	1.026	0.997	0.998	1.029	0.999

Abbreviations: EEPC: energy and environmental productivity change; EC: efficiency change; PEC: pure technical efficiency; TC: technological change; SEEC: scale effect on efficiency change.

**Table 4:** Cumulative value of EEPC and its decomposition for classification by resource-dependence.

RD	Year	2004	2006	2008	2010	2012	2014	2016	2018	Growth	Contribution
Low	EEPC	0.900	0.930	1.022	1.108	1.139	1.131	1.303	1.358	2.06%	100.00%
	TC	0.893	0.930	1.038	1.129	1.120	1.185	1.359	1.421	2.37%	115.02%
	EC	1.007	1.000	0.985	0.981	1.017	0.954	0.959	0.956	−0.30%	−14.67%
	PEC	1.016	1.008	0.991	1.007	1.038	0.973	0.961	0.959	−0.28%	−13.47%
	SEC	0.992	0.992	0.994	0.974	0.980	0.981	0.998	0.996	−0.02%	−1.21%
Medium	EEPC	0.917	0.947	1.044	1.060	1.111	1.127	1.325	1.433	2.43%	100.00%
	TC	0.915	0.931	1.021	1.094	1.089	1.179	1.355	1.423	2.38%	98.18%
	EC	1.003	1.017	1.023	0.969	1.020	0.956	0.978	1.007	0.04%	1.78%
	PEC	1.019	1.017	1.026	1.001	1.036	0.980	0.996	1.002	0.01%	0.46%
	SEC	0.984	1.000	0.997	0.967	0.985	0.975	0.982	1.005	0.03%	1.32%
High	EEPC	0.955	0.990	1.062	1.099	1.122	1.111	1.279	1.398	2.26%	100.00%
	TC	0.942	0.964	1.048	1.133	1.156	1.255	1.445	1.500	2.74%	121.31%
	EC	1.014	1.027	1.013	0.971	0.971	0.885	0.885	0.932	−0.47%	−20.74%
	PEC	1.013	1.025	1.008	1.002	0.967	0.913	0.923	0.953	−0.32%	−14.19%
	SEC	1.001	1.001	1.005	0.969	1.004	0.970	0.958	0.978	−0.15%	−6.57%

**Table 5:** Cumulative value of EEPC and its decomposition for classification by region.

Region	Year	2004	2006	2008	2010	2012	2014	2016	2018	Growth	Contribution
Eastern	EEPC	0.896	0.971	1.115	1.187	1.199	1.191	1.331	1.447	2.49%	100.00%
	TC	0.931	1.002	1.179	1.280	1.246	1.290	1.464	1.532	2.89%	115.81%
	EC	0.963	0.969	0.946	0.928	0.962	0.923	0.909	0.944	−0.38%	−15.36%
	PEC	0.987	0.963	0.961	0.957	0.974	0.942	0.935	0.936	−0.44%	−17.66%
	SEC	0.976	1.006	0.984	0.969	0.988	0.979	0.972	1.009	0.06%	2.31%
Northeastern	EEPC	0.951	0.984	1.106	1.184	1.210	1.259	1.444	1.557	3.00%	100.00%
	TC	0.936	0.985	1.096	1.196	1.205	1.339	1.460	1.643	3.37%	112.30%
	EC	1.016	0.998	1.009	0.990	1.004	0.940	0.989	0.948	−0.36%	−11.90%
	PEC	1.015	0.990	1.020	1.028	1.004	0.982	1.044	0.998	−0.01%	−0.38%
	SEC	1.000	1.008	0.989	0.963	1.001	0.958	0.948	0.949	−0.35%	−11.52%
Western	EEPC	0.937	0.958	1.033	1.057	1.106	1.114	1.311	1.370	2.12%	100.00%
	TC	0.916	0.905	0.979	1.064	1.064	1.143	1.344	1.368	2.11%	99.70%
	EC	1.023	1.059	1.055	0.993	1.040	0.974	0.975	1.001	0.01%	0.29%
	PEC	1.029	1.062	1.065	1.035	1.061	1.030	1.022	1.025	0.16%	7.68%
	SEC	0.995	0.997	0.991	0.960	0.980	0.945	0.954	0.977	−0.16%	−7.37%
Middle	EEPC	0.922	0.939	0.999	1.031	1.069	1.053	1.236	1.351	2.03%	100.00%
	TC	0.908	0.931	0.995	1.060	1.080	1.177	1.361	1.415	2.34%	115.42%
	EC	1.015	1.009	1.003	0.973	0.990	0.894	0.908	0.955	−0.31%	−15.07%
	PEC	1.020	1.018	0.987	0.991	0.993	0.889	0.895	0.940	−0.41%	−20.24%
	SEC	0.995	0.991	1.016	0.981	0.996	1.006	1.014	1.016	0.11%	5.19%

**Table 6:** Classification of resource-based cities based on static efficiency and dynamic productivity change.

Group	City
HH (24)	Anshan, Chifeng, Daqing, Dongying, Handan, Hengyang, Huludao, Jixi, Jining, Liaoyuan, Luzhou, Luyang, Nanchong, Nanyang, Puyang, Sanmenxia, Songyuan, Tai’An, Tonghua, Xianyang, Xuzhou, Ya’An, Zaozhuang, Zibo
HL (27)	Baoshan, Bozhou, Chuzhou, Dazhou, Ordos, Ganzhou, Guangan, Hechi, Hulun Buir, Huzhou, Lincang, Linyi, Longyan, Longnan, Nanping, Pu’Er, Qingyang, Sanming, Shaoyang, Weinan, Suqian, Suzhou, Xuancheng, Yan’An, Yichun, Yunfu, Zhaotong
LH (31)	Baiyin, Baotou, Baoji, Benxi, Chengde, Datong, Ezhou, Fushun, Fuxin, Guangyuan, Hebi, Huaibei, Huainan, Huangshi, Jiaozuo, Laiwu, Liupanshui, Loudi, Ma’Anshan, Panzhihua, Panjin, Pingdingshan, Pingxiang, Shaoguan, Tangshan, Tongchuan, Tongling, Wuhai, Xinyu, Yangquan, Zhangye
LL (25)	Anshun, Baise, Chenzhou, Chizhou, Hezhou, Hegang, Jinchang, Jincheng, Jinzhong, Kalamay, Linfen, Lvliang, Pingliang, Qitaihe, Qujing, Shizuishan, Shuangyashan, Shuozhou, Wuwei, Xinzhou, Xingtai, Yulin, Yuncheng, Zhangjiakou, Changzhi
